# Improving Packet Delivery Performance of Publish/Subscribe Protocols in Wireless Sensor Networks

**DOI:** 10.3390/s130100648

**Published:** 2013-01-04

**Authors:** Ernesto García Davis, Anna Calveras, Ilker Demirkol

**Affiliations:** Wireless Network Group (WNG), Universitat Politècnica de Catalunya, C/Jordi Girona 1-3 Mòdul C3, 08034 Barcelona, Spain; E-Mails: anna.calveras@entel.upc.edu (A.C.); ilker.demirkol@entel.upc.edu (I.D.)

**Keywords:** publish/subscribe, reliability, CoAP, MQTT-S, wireless sensor networks, retransmission timeout

## Abstract

MQTT-S and CoAP are two protocols able to use the publish/subscribe model in Wireless Sensor Networks (WSNs). The high scalability provided by the publish/subscribe model may incur a high packet loss and therefore requires an efficient reliability mechanism to cope with this situation. The reliability mechanism of MQTT-S and CoAP employs a method which defines a fixed value for the retransmission timeout (RTO). This article argues that this method is not efficient for deploying publish/subscribe in WSN, because it may be unable to recover a packet, therefore resulting in a lower packet delivery ratio (PDR) at the subscriber nodes. This article proposes and evaluates an adaptive RTO method, which consists in using a Smooth Round-trip Time and multiplying it by a constant parameter (K). Thanks to this method, the reliability mechanism of MQTT-S and CoAP would be able to react properly to packet loss and would also be lightweight in terms of energy, memory and computing for sensor nodes where these resources are critical. We present a detailed evaluation of the effects of the K value on the calculation of the adaptive RTO method. We also establish the setting for obtaining the highest PDR on the subscriber nodes for single-hop and multi-hop scenarios. The results for single-hop scenario show that use of the appropriate K value for the adaptive RTO method increases the PDR up to 76% for MQTT-S and up to 38% for CoAP when compared with the use of fixed RTO method for both protocols, respectively. Meanwhile the same comparison for multi-hop scenario, the adaptive RTO method increases the PDR up to 36% for MQTT-S and up to 14% for CoAP.

## 1. Introduction

Wireless sensor networks (WSNs) have been deployed to measure or detect physical and environmental events such as temperature, pressure, humidity and pollution levels, or any critical parameters of a given scenario. Applications usually send queries to sensor nodes to retrieve values periodically from the measurements or detections. This type of data collection method is called polling. However, recent applications of WSN, such as home automation, industry process control, environment monitoring, smart grid and ambient assisted living, require information when an event of interest occurs in order to react in real-time.

The polling method is not efficient for this type of application, since the sensor nodes have to be queried continuously. This situation leads to a waste of valuable resources such as energy, processing and bandwidth, which are very limited in these kinds of devices and networks. Moreover, current trends such as the Internet of Things (IoT) propose that devices should be connected and able to sense and communicate data from their surroundings. This is expected to result in networks with much higher node densities. Thus, it is important to use an efficient and scalable method to gather information from networks with high densities.

The problem described above could be overcome using the publish/subscribe model [1]. This consists of entities called subscribers who have the ability to express their interest in events produced by other entities called publishers. This process of registering interest in an event is called subscription. On the other hand, publishers are entities that generate certain information by publishing their own information. The strength of this model is based on decoupling in time; that is, publishers and subscribers do not need to be actively participating in the interaction at the same time. Furthermore, the model is decoupled in space, *i.e.*, publishers and subscribers do not need to know about each other. Moreover, publishers and subscribers can produce or consume events in an asynchronous way. In other words, subscribers can be asynchronously notified of an event while simultaneously performing some activity and publishers for their part are not blocked while producing events. This decoupling of publishers and subscribers allows greater scalability and flexibility as well as providing a more dynamic network topology. These are desirable features for WSN and for current trends such as IoT, and make the publish/subscribe model highly suitable for this purpose.

Certain publish/subscribe protocols have already been proposed for wireless sensor networks [2–4], most of which however are customized protocols, *i.e.*, either they are built for a specific application and without standardization in the communication protocol or their development is no longer supported.

Among these protocols, Message Queuing Telemetry Transport [4] (MQTT-S) is the most highly developed and the one with most attempts at standardization [5]. In fact, this protocol has been widely used for remote monitoring applications [6,7], messaging applications [8] and a range of home automation applications. It can be considered as a version of MQTT [9] adapted to the peculiarities of a wireless communication environment.

On the other hand, work is being carried out to enable the use of standard and well-known protocols for interaction with WSNs. The use of web services on the Internet has become ubiquitous in most applications, and depends on the fundamental Representational State Transfer (REST) architecture of the web [10]. There is a work-in-progress protocol from the IETF CORE group [11] known as CoAP [12]. CoAP is the RESTful protocol in constrained sensor networks that aims at realizing the REST architecture in a suitable form for the most constrained nodes (e.g., 8-bit microcontrollers with limited RAM and ROM) and networks such as WSNs. In addition to the web functionality, CoAP also provides a publish/subscribe model, which allows subscription to the events of interest published for sensor nodes.

There are several studies on the use of reliable transport protocols in WSNs [13–15], however MQTT-S and CoAP are application protocols that have been designed to work over unreliable datagram services such as the User Datagram Protocol (UDP). Therefore they provide mechanisms to ensure the data reliability. Moreover, when we refer to the reliability mechanisms of the application layer we mean in general the reliability mechanisms provided by MQTT-S and CoAP. Hence, in this paper, we study the reliability at the application layer of both protocols.

The reliability mechanisms for both protocols use a method which we refer to in this paper as the fixed retransmission timeout (RTO) method. It establishes a fixed value for the RTO instead of considering the network conditions as a parameter for calculating its value. We argue that the fixed RTO method is inefficient when delivering data in a reliable way in all situations, since the dynamic topology and the density of WSN nodes may affect the network conditions in terms of the round trip time (RTT) of a packet.

While in terms of computational process a fixed RTO method is simpler than maintaining an estimate of the RTT for all destinations, this technique does have certain drawbacks. First of all, a short RTO value could give rise to spurious retransmissions, which means that unnecessary retransmissions might be performed in cases where the previous message is still in transit. This situation leads to a waste of bandwidth, energy and computation, all of which are valuable resources in networks such as WSN. Secondly, a larger RTO value may lead to a slow or late reaction to the loss of packets, which in turn results in an increase in the perceived delay and a decrease in the packet delivery ratio (PDR) at the subscriber node.

Furthermore, MQTT-S and CoAP have different mechanisms to discard the sending of publication messages, which we refer to as the publication discipline. The publication discipline is activated when there is a pending confirmation of a publication message, and a new publication is generated. The difference between the publication discipline of MQTT-S and CoAP is that the former discards the new publication message and the latter the old one. The choice of publication disciplines could affect the PDR of subscriber nodes, because the publication messages are discarded at the Application layer (MQTT-S or CoAP) of publisher nodes.

We realize that the RTO value is one of the factors that could directly affect the number of discarded publication messages for these publication disciplines. The use of a fixed RTO method could set an inappropriate value for the RTT, which in turn would result in a higher probability of a new publication message being generated while the RTO is active. Consequently, the publication discipline would discard the publication message.

For all these reasons, the fixed RTO is not efficient for the aforementioned applications. We focus on one-hop scenarios and multi-hop scenario that is generally in line with both building automation and industrial monitoring and control [16] applications. In this case, devices exist in the application area that act as subscriber nodes and need to receive a certain type of information for control purposes. Further devices consist of publisher nodes in charge of sending this information. Communication between these devices is in many cases carried out through a device acting as the central controller or gateway device. If in this kind of application the reliability requirement is not met, the correct execution of control actions may be severely compromised. It therefore becomes vital to evaluate and optimize the value of RTO in order to adapt it to the network conditions. In this way we could gain a higher PDR, thereby reducing the number of discarded publication messages, preventing spurious retransmissions and reacting properly to recover packet loss.

We propose to use an approach to calculate the RTO using Smooth Round Trip Time (SRTT) [17] as a metric for the network condition, multiplied by a K parameter for MQTT-S and CoAP. We investigate the effect of the K parameter for the RTO calculation, and show that by setting an appropriate K value, much higher PDR can be obtained for subscriber nodes compared to the fixed RTO approach defined by MQTT-S and CoAP. We also evaluate other metrics such as the discarded publication message ratio, the retransmitted message ratio and the duplicated message ratio in order to demonstrate why the K value we found is suitable for the efficient setting of RTO for the investigated scenarios.

Furthermore, we study the effect of using the reliability mechanism employed at the MAC layer, namely the use of MAC Acknowledgments. We show the scenarios in which MAC Acknowledgements are useful for delivering reliable data, together with the application reliability provided by MQTT-S and CoAP protocols.

The remainder of this article is structured as follows: Section 2 describes the main features and design aspects of MQTT-S and CoAP. A comparison and discussion of both protocols, focused mainly on their reliability mechanisms and publication disciplines, is presented in Section 3. Section 4 describes the setup of the simulations that we use to evaluate the proposed reliability mechanism for both protocols. The results obtained by simulation for single-hop and multi-hop scenarios are shown in Section 5. Finally, in Section 6 we conclude our work and present future research lines.

## 2. Background

In this section, we describe the two publish/subscribe protocols focused on the wireless sensor networks already mentioned in the previous section. We focus mainly on the reliability mechanism and the publication discipline of both protocols.

### 2.1. Message Queuing Telemetry Transport (MQTT-S)

MQTT-S protocol is considered to be an extension of the publish/subscribe protocol Message Queuing Telemetry Transport (MQTT) [9]. MQTT-S is optimized for the implementation on low-cost, battery-operated devices with limited processing and storage resources such as wireless sensor devices.

The design principles are aimed at minimizing network bandwidth and device resource requirements whilst targeting reliability. These principles also make the protocol suitable for machine-to-machine (M2M) or the Internet of Things world of connected devices as well as for mobile applications where bandwidth and battery power are at a premium.

**Figure 1.**
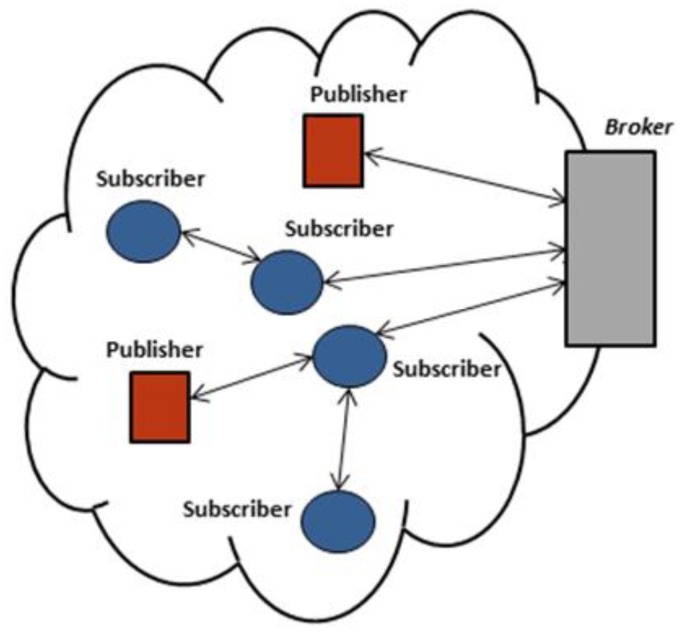
General MQTT-S architecture.

Figure 1 shows the three components of the MQTT-S architecture. A broker node is responsible for managing subscriptions as well as storing and sending publications to corresponding WSN subscriber nodes. In addition, the broker is able to provide the same services to nodes in external networks (Internet) by using a gateway node, This node is located at the edge of the WSN and is in charge of message mapping between the MQTT (for external network) and MQTT-S (for WSN) protocols. A MQTT-S gateway may or may not be integrated within the broker. The sensor nodes of WSN are able to act as publisher, subscriber or relay nodes in case of a multi-hop scenario, in order to establish connection with the broker node.

Furthermore, MQTT-S can operate on any network technology that provides a datagram service. MQTT-S does not require the connection oriented transport provided by Transport Control Protocol (TCP), so it is well-suited for use over UDP.

#### 2.1.1. Reliability

MQTT-S provides a reliability mechanism consisting of three QoS levels. Level 0 offers a best-effort delivery service, and no retransmission or Acknowledgements are defined. Level 1 allows the retransmission of messages until they are acknowledged by the receivers; however, this QoS level does not prevent the messages from arriving at the destination multiple times because of retransmissions, which is referred as duplicate reception. Finally, Level 2 ensures not only the reception of messages, but also that they are delivered only once to the destination. This is achieved through a four message handshake.

Since QoS Levels 1 and 2 require a retransmission mechanism, MQTT-S defines two parameters in its best-practice guidelines [18]: A fixed RTO (10–15 s) and a maximum retransmission number (3–5 s). Since there is no reference to an application scenario in these best-practice guidelines, we use the lower and upper values of the RTO for evaluation purposes.

#### 2.1.2. Publication Discipline

MQTT-S uses a “stop and wait” mechanism for the transmissions of publication messages with QoS Levels 1 and 2. This means that any point in time a publisher node has to wait for the termination of its publication message flow with the broker node before it can start a new one. A publication message flow finishes when the publisher node receives the corresponding confirmation message according to the QoS level. This situation is repeated between the broker node and the subscriber node.

According to this way of working, a publication discipline for QoS Levels 1 and 2 should be provided to handle the publication messages generated while a publish message flow is in progress. It should be pointed out that the RTO is active while the publication message flow is in progress. The MQTT-S specification [15] does not indicate the way to handle this situation. One way to address this issue would be for the publisher node to queue the new message publication until the confirmation message is received from the current publication message flow. However, due to the limited memory resources of sensor nodes, this aspect becomes a critical operational factor. Accordingly, in the experiments carried out to evaluate this publication discipline we do not consider the queuing mechanism. Instead, if there is a pending confirmation of a previous publication message, the new publication message is discarded. We refer to this discipline as the “persistent mode” discipline, because it always attempts to retransmit the earlier publication message and receive the confirmation message instead of sending the new one. Discussion of these mechanisms and their comparison with those of the CoAP protocol is addressed in the following sections.

### 2.2. Constrained Application Protocol (CoAP)

CoAP is a Representational State Transfer (RESTful) protocol optimized for resource constrained networks and targets the Internet of Things and M2M applications such as smart grid, and building and home automation. It is based on a REST architecture in which resources are made available by an application process and identified by Universal Resource Identifiers (URI). It is built on top of the UDP and therefore has a significantly lower overhead than Transmission Control Protocol (TCP).

In addition, CoAP defines two kinds of interactions between end-points: (1) The client/server interaction model, where request messages initiate a transaction with a server, which may send a response to the client with a matching transaction ID, and (2) A publish/subscribe interaction model called the observer model [19], where a server (publisher node) can send notify messages (publications) to an observer (subscriber node) about a resource (event) that the subscriber is interested in receiving.

**Figure 2.**
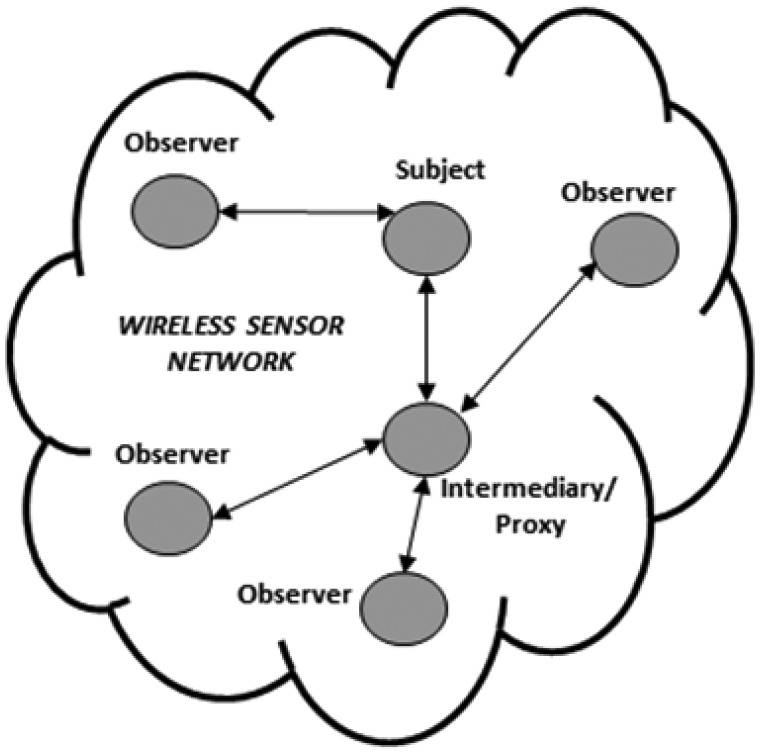
Architecture of the CoAP observer model.

The client/server interaction model is based on the polling method. As mentioned earlier, this method is unsuitable for applications requiring information in real-time in order to react when an event of interest occurs. Therefore, we focus only on the publish/subscribe interaction model provided by CoAP, which is illustrated in Figure 2. With this model, CoAP allows a subscriber constantly to observe the events. This is done by the subscriber registering its interest in the event by means of an extended GET request sent to the publisher node. The publisher node establishes an observation relationship between the subscriber and the event, *i.e.*, subscription is performed. The publisher notifies each subscriber node that has an observation relationship with the event.

Although in the general architecture of this model the publisher node also plays the role of broker, CoAP also enables high scalability and efficiency through a more complex architecture, which in fact supports the use of caches and intermediaries (proxy) nodes that multiplex the interest of multiple subscribers in the same event into a single association, as shown in Figure 2.

#### 2.2.1. Reliability

CoAP provides a reliability mechanism through two types of messages: (1) A Confirmable (CON) message: The publication message is retransmitted if no delivery acknowledgement was received, (2) A Non-Confirmable (NON) message: In this case there is no need to acknowledge the message.

In the case of CON messages, the reliability mechanism uses an RTO fixed method. This consists of setting an initial RTO value to a random number between an ACK TIMEOUT constant and an ACK TIMEOUT multiplied by ACK_RANDOM_FACTOR constant [12]. The messages that have not been acknowledged within RTO duration are retransmitted and subsequently this RTO value is doubled (exponential back-off mechanism).

CoAP also defines a MAX_RETRANSMIT constant, which specifies the maximum number of message retransmissions. Table 1 shows the corresponding values for each of these protocol constants that are defined in [12].

Compared with MQTT-S, we are able to state that Non-confirmable CoAP messages correspond to messages of QoS Level 0 in MQTT-S and Confirmable messages are similar to QoS Level 1. Since the CoAP protocol has only two types of QoS, the QoS Level 2 of MQTT-S is not evaluated in this article.

#### 2.2.2. Publication Discipline

CoAP also uses a “stop and wait” mechanism for the transmission of CON messages. Therefore, in a similar manner to MQTT-S, this protocol also requires a publication discipline to handle publication messages generated while the publication message flow is in progress (RTO is active). In this context, the publication discipline of CoAP will be activated when the publisher node wants to notify the subscriber node of a change in the state of the event. Thus, it must stop the retransmission of previous publication message and transmit the new one with the number of attempts remaining from the previous publication message.

## 3. Comparison and Discussion of MQTT-S and CoAP

As mentioned above, MQTT-S and CoAP protocols use a fixed value for RTO calculation. It could be feasible for deployments where the RTT is close to the defined RTO value. Nevertheless, a fixed RTO value is not compatible with the scalability and flexibility features provided by the publish/subscribe model on WSN. Due to changes in network conditions, a fixed RTO could lead to spurious retransmissions if its value is too short, resulting in a waste of resources. Otherwise, it would cause the reliability mechanism to react too late to recover the packet loss.

The authors in [20] propose computation of the RTO value in the same way as with RFC 6298 [17]. This defines an adaptive RTO method using an algorithm to compute a smoothed RTT (SRTT) and another algorithm to calculate an RTT variance (RTTVAR). We argue that this method is effective for obtaining a proper RTO value for the network conditions, although it is not effective for sensor nodes. The use of these two algorithms means that the state for each destination at the sender must be maintained, which may require high amount of resources in terms of memory and computing, which in turn results in more energy consumption in the sensor nodes.

We consider using the algorithm to obtain an estimated SRTT. Furthermore, in order to ensure a good estimation of RTO, we propose the multiplication of SRTT by a K factor, but not by using the RTTVAR. The RTO calculation is therefore performed by multiplying the estimated SRTT by a K parameter:

(1) RTO=SRTT×K

where SRTT [17] is given by:

(2) SRTT=(1−α)×SRTT+α×RTT

where α = 1/8 and RTT is the time from when a publication message is generated and the confirmation message is received at the application level.

This method would lead to a reduction in the process of computing RTO in the sensor nodes. Since nodes in WSN are constrained-resources devices, a lightweight computing would help to reduce energy waste and extend the lifetime of the node in the network.

Another situation in which the way of calculating the RTO plays an important role is in the publication discipline of MQTT-S and CoAP protocols. The publication discipline of both protocols has a direct impact on the number of discarded publication messages. This is because the publication discipline will discard publication messages if a new publication messages is received while the RTO is activated.

It should be noted that publisher nodes always discard a greater number of publication messages in comparison with the broker node. This is because the publisher nodes are in charge of publication message generation. Thus, if a confirmation message is not received before a publication message is generated; the publication discipline discards the publication message. The reasons why they have not received this confirmation could be:

- Because the broker has not received the publication message, thus requiring a retransmission from the publisher nodes.
- Because the broker has received the publication message, but the confirmation message is lost. Likewise in this case the publication from the publisher could also be retransmitted, but it will become a duplicate publication for broker node.

In both cases, the discarded ratio of publications is proportional to the number of publisher nodes. In other words, as we increase the number of publishers, the discarded rate of publications increases. This is because a greater number of publisher nodes in the network may give rise to two situations: increased contention for access to the channel and a higher probability of collision, resulting in a greater delay in channel access. Both situations cause application layer (CoAP or MQTT-S) retransmissions, and increase the probability of generating a new publication while waiting for a confirmation of a previous publication (RTO timer is activated).

As mentioned above, a fixed RTO value is unable to respond to changing network conditions; thus for larger values of RTO, the number of discarded publication messages could be high. As a consequence, the packet delivery ratio of subscriber nodes will also decrease.

The publication discipline of both protocols differs in the way it discards the publication messages. On one hand, MQTT-S attempts a persistent delivery of a generated publication to the subscriber. This means that it discards a new publication if there is a pending confirmation of a previous publication. On the other hand, CoAP always attempts to deliver a new publication message. This means that a new publication is sent and the retransmission of the old publication is discarded (canceled) should it be needed.

Therefore, the effect of the RTO value on the publication discipline of MQTT-S causes the discarding of new publication messages, which in turn results in the loss of new data in the subscriber node. In contrast, the effect on the publication discipline of CoAP is reflected in the cancellation of possible retransmissions of old publication messages with confirmable messages. As a consequence, the lost publication messages would be treated as a publication with a Non-Confirmable message, which in turn causes a decrease in the packet delivery ratio at the subscriber nodes. This is because MQTT-S could be oriented to applications where the delivery of most of the data is required. In contrast, CoAP attempts to keep the subscriber node abreast of the most recent data from an event. The goodness of the MQTT-S and CoAP publication discipline depends on the application area and is beyond the scope of this article.

An adaptive RTO that takes network conditions into account would reduce the number of dropped publication messages for both protocols. However, the decrease in the number of discarded publications will also depend on the publication generation rate. This situation is beyond the control of an adaptive RTO.

## 4. Performance Evaluation

In this section we describe the simulation environment and performance metrics defined to evaluate both protocols.

### 4.1. Simulation Environment

Simulation experiments were carried out using OMNet [21]. In this article, we focus on single hop and multi-hop network topology, as previously illustrated in Figures 1 and 2. We consider a potential application scenario of industrial automation. In this context, the goal of the application is the monitoring and control of critical parameters in a warehouse through a WSN deployment.

Two types of devices are deployed in different parts of the application area: publisher and subscriber nodes. The publisher nodes are responsible for measuring the critical parameters in the warehouse. Additionally, there are two subscriber nodes, one of which receives publication messages in a best-effort mode for the monitoring process only. This means that the publication messages are received using the QoS Level 0 for MQTT-S, and NON messages for CoAP. The other subscriber node receives publication messages in reliable mode for controlling the critical parameters. This means that it receives publication messages using QoS Level 1 (MQTT-S) or CON (CoAP). When the critical parameter is above a predefined threshold, this subscriber node acts appropriately. For instance, activating an alarm to evacuate the personnel or by turning on the ventilation system to avoid a risky situation.

For this critical application where the monitoring and early detection of critical condition is crucial, we consider that the data is generated periodically every second by publisher nodes and is then sent to the broker node. We also choose this data generation rate to study the system in stress condition. Since each subscriber node receives the publication messages with a different reliability level, the broker node has to receive the publication messages from publisher nodes with the maximum reliability level, as previously explained.

The number of publisher nodes varies 10–100 in steps of 10. We use the term “broker” node to refer to the central node specified in the MQTT-S architecture and the role of the proxy node for CoAP protocol.

**Figure 3.**
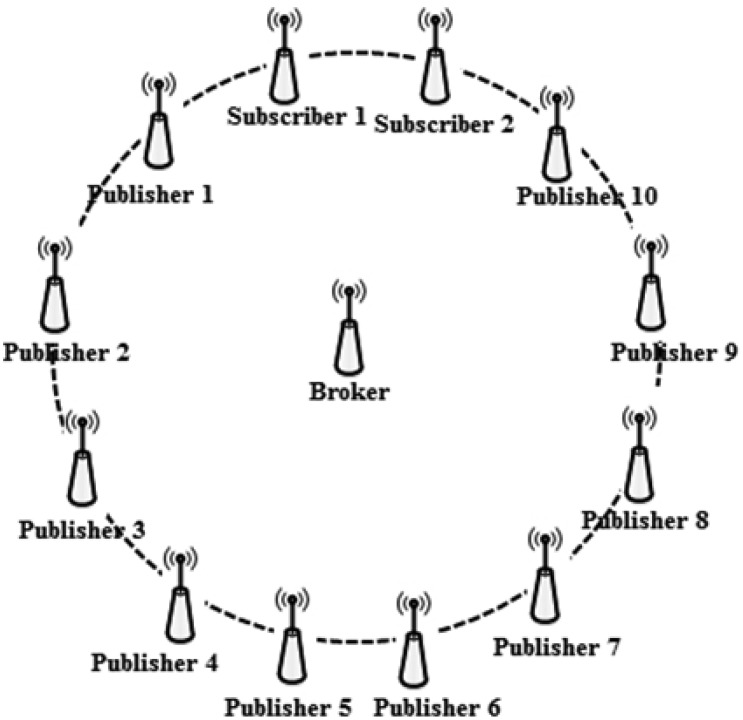
Simulated network topology.

We consider several single hop and multi-hop scenarios as follows. In the single hop scenario all nodes are within communication range of each other. Subscribers and publisher nodes are placed at the same distance from the broker to achieve fairness among nodes by preventing the capture effect, as illustrated in Figure 3.

Besides, we extend the single hop scenario to build a distributed system based on multiple broker nodes, by using the publish/subscribe model. This allows the extension of the coverage area of the application and the communication of interested parties (sensors and actuators) located in more than one hop distance of each other. Publication messages originated in publisher nodes located from more than one hop away are received through the broker node to which the subscriber nodes are connected. This is possible because of the broker node intercommunication. That is, broker node subscribes on behalf of its subscriber nodes to another broker node that the publisher node with information of interest connected to.

On the other hand, the multi-hop scenario consists of nodes that are located up to 3 hops from the broker node. The messages originating from a publisher node are routed to the broker node through multiple nodes and the broker node has to route the messages in a similar way to the subscriber nodes. Static routes are defined for the sake of the simplicity of the simulation environments.

In the PHY layer, we use the 2.4 GHz range with a bandwidth of 250 kbps based on IEEE 802.15.4 [22]. In addition, the maximum number of MAC-layer retransmissions is 3, which is the default value of IEEE 802.15.4 [22]. For energy consumption calculation, we use the energy model provided by the simulator [21] with the power consumption settings based on the TelosB datasheet [23].

Each simulation experiment lasts for 500 s. Each point in the graph presented in this section is based on the average of ten simulation runs. A confidence interval with probability of 95% has been calculated for the PDR metric of each scenario as the major metric of the paper. Table 2 summarizes the parameters and their assigned values used in the simulation.

### 4.2. Performance Metrics

In order to evaluate the reliability mechanism of MQTT-S and CoAP protocols, we consider the packet delivery ratio, the dropped publication ratio, the retransmitted publication ratio and the duplicated publication ratio as performance metrics.

- **Packet Delivery Ratio (PDR):** This is a crucial metric for evaluating the performance of the reliability mechanism of MQTT-S and CoAP protocols. It expresses the ratio of the total number of publication messages received by each subscriber node, up to the total number of publication messages generated by all publisher nodes of the events to which the subscriber node has subscribed. It does not take into account duplicated publication messages received by subscriber nodes.
- **Discarded Publications Ratio (DPR):** With this metric we evaluate the impact of the publication discipline in the PDR. This is because the discarded publications will never be received by the subscriber node, so the PDR will decrease as the DPR increases. This measure is the ratio of the number of discarded publication messages (at the publisher nodes or at the broker), and the amount of messages generated (at the publisher nodes).
- **Retransmitted Publications Ratio:** This is an important metric used to evaluate the effect of the RTO value, because a good RTO value should reduce spurious retransmissions as well as ensuring reaction without delay in the case of message losses. It is the ratio of the total number of publication messages retransmitted to the total number of sent publications messages. This metric is evaluated for the total number of publisher nodes and for the broker node.
- **Duplicated Publications Ratio:** This indicates the ratio of the number of duplicated publication messages received to the total number of publication messages received. We evaluate this metric in the broker node and in the subscriber node with QoS1 (for MQTT-S) and subscriber node with CON (for CoAP). It counts the number of retransmitted publications that reach the broker and the subscriber nodes.

Each of the above metrics was investigated by varying the number of publisher nodes and the K value used for RTO calculation, as previously explained. Furthermore, we investigate the effect of using or not using MAC Acknowledgements in order to find situations in which this metric may be needed.

## 5. Results and Discussion

In this Section, we analyze and discuss the results obtained from the evaluation of MQTT-S and CoAP protocols by simulation using our adaptive method. We investigate the value of K from which we obtain the highest PDR for the subscriber node with QoS1 for MQTT-S and the confirmable (CON) message for CoAP, respectively. Furthermore, in order to evaluate in what way the adaptive RTO method is better than the one used by MQTT-S and CoAP, we compare their PDR values. Finally, we evaluate the other metrics such as: discarded publication ratio, retransmitted publication ratio, duplicated publication ratio and energy consumption, in order to determine why the highest PDR is obtained with a specific K value.

### 5.1. Single Hop Scenario

In this section, we show and discuss the results obtained for the single hop and extended single-hop scenarios that we have described previously.

#### 5.1.1. Packet Delivery Ratio (PDR)

The PDR is affected by the number of publisher nodes. As the number of publisher nodes increases, a lower PDR is obtained. As expected, the probability of success in accessing the medium decreases when a greater number of nodes contend for access to the channel. We evaluate the effect of the K value with our proposed method for RTO calculation, as well as the effect of using or not using MAC Acknowledgements. The aim of this evaluation is to find the appropriate K value for obtaining the highest PDR for subscriber nodes for MQTT-S and CoAP.

In general, the subscriber nodes achieve a higher PDR as the K value increases for MQTT-S and CoAP. One of the reasons for this is because spurious retransmissions are reduced. However, we found that for a value above a specific K value the PDR of subscriber nodes begins to decrease. On the other hand, when considering the effect of using or not using MAC Acknowledgements, both protocols show a similar behavior.

**Figure 4.**
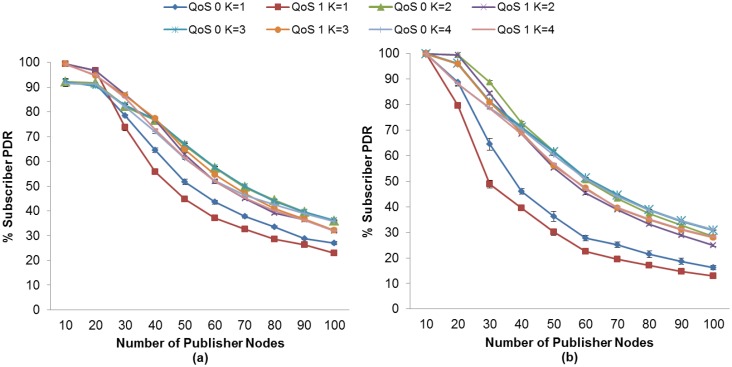
Effect of the number of Publisher Nodes on the Subscribers PDR depending of the K value of RTO with MQTT-S (**a**) without and (**b**) with MAC Acknowledgements.

Firstly, for MQTT-S, with our proposed method, we observe that the highest PDR for the subscriber node with QoS 0 is obtained with K = 3, without the use of MAC Acknowledgements, as can be seen in Figure 4(a). However, Figure 4(b) shows that for a number of publisher nodes less than 40, the highest PDR is obtained by using MAC Acknowledgements with K = 2.

In this case, the value of K = 2 enables the MAC Acknowledgements to recover most of the lost messages before MQTT-S retransmissions are activated from publisher nodes. Otherwise, a number of publisher nodes greater than 40 when using MAC Acknowledgements results in a higher probability of collision and loss of publication messages, and thus in an increase in delay. In this context, the value of K = 2 would lead to spurious retransmissions because the MQTT-S retransmissions would be activated before MAC Acknowledgements attempt to recover the lost messages. This situation results in a low PDR for this subscriber node.

Note that the PDR of a subscriber with QoS 1 changes from 30 publisher nodes onwards, unlike the case of the PDR of a subscriber with QoS 0, where the PDR changes from 40 publisher nodes onwards. This is because the additional messages used on the reliability mechanism provided by QoS Level 1 of MQTT-S congest faster with a number of nodes greater than 30 publisher nodes.

A similar situation occurs for the subscriber node with QoS 1.The highest PDR is obtained with K = 3 without the use of MAC Acknowledgements, as shown in Figure 4(a). An exception occurs for a number of publisher nodes less than 30; in this case the subscriber node with QoS 1 obtains the highest PDR employing K = 2 with the use of MAC Acknowledgements, as shown in Figure 4(b).

**Figure 5.**
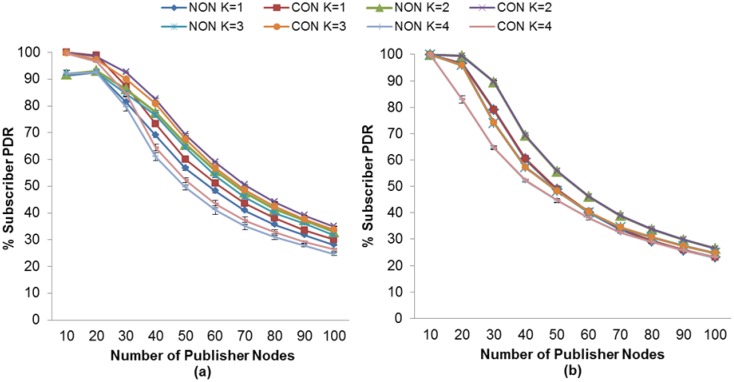
Effect of the number of Publisher Nodes on the Subscribers PDR depending of the K value of RTO with CoAP without (**a**) and with (**b**) MAC Acknowledgements.

For CoAP, both subscriber nodes obtain the highest PDR with K = 2, without the use of MAC Acknowledgements, as can be seen in Figure 5(a). Nevertheless, Figure 5(b) shows that for a number of publisher nodes less than 40, the use of MAC Acknowledgements is required for obtaining the highest PDR for a subscriber with NON messages. The same situation applies for the subscriber with CON messages. In this case, the use of MAC Acknowledgements is required for a number of publisher nodes less than 30 in order to obtain the highest PDR. The reasons for this are the same as those previously explained for the subscriber node with QoS 0 using the MQTT-S protocol with our proposed RTO method.

Another important aspect to take into account in the PDR is the effect of the discarded publication ratio (DPR) caused by the publication discipline of each protocol, which is discussed in detail in the following section. For a higher DPR, a lower PDR for each subscriber node is obtained. For the publication discipline of the MQTT-S protocol, a fraction of the publications generated by the publisher nodes or received by the broker node are discarded before being sent to the subscriber nodes. This fraction is not sent to the channel, and therefore reduces the number of publication messages received by the subscriber nodes. In the case of the CoAP publication discipline, neither the publisher nodes nor the broker node will retransmit a publication message pending confirmation if it generates or receives a new publication. This means that the publication messages will not be recovered in case of losses, and consequently the PDR of subscriber nodes is reduced.

In summary, one may observe that the publication discipline of CoAP leads to a PDR for the subscriber node with reliable delivery (QoS 1 and CON messages, respectively) that is higher than the publication discipline used by MQTT-S. Recall that CoAP publication discipline gives priority to sending the new publications rather than attempting to retransmit the old one as MQTT-S does. This situation increases the PDR in the case where the publication messages have been received by the subscriber node but the confirmation is lost.

**Figure 6.**
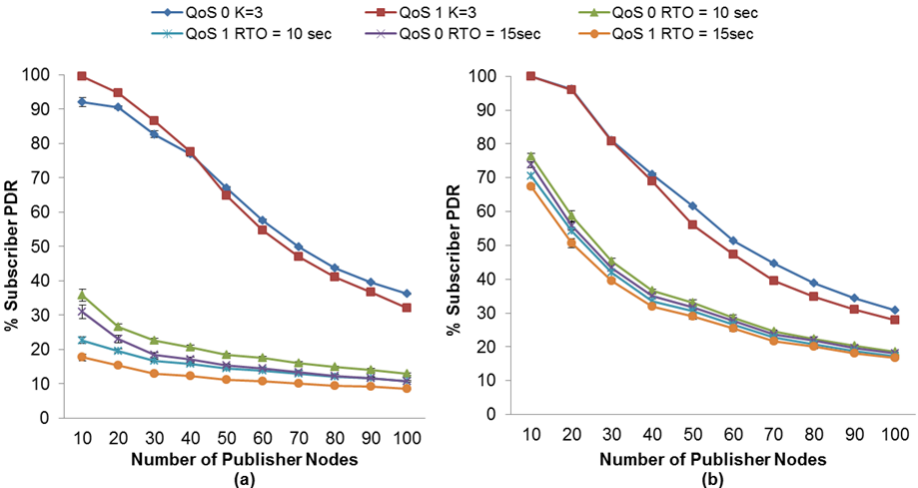
PDR comparison between the RTO method of MQTT-S calculation and our proposal without (**a**) and with (**b**) MAC Acknowledgements.

In addition, we compare the fixed RTO method used by MQTT-S with our proposal. In Figure 6(a), one may observe that when MAC Acknowledgements are not used, subscriber nodes obtain a PDR with the fixed RTO method used by MQTT-S that is lower than when using our proposal. As expected, the fixed RTO values of 10 and 15 s cause the MQTT-S retransmissions to be activated too late to recover the message losses. The advantage of the RTO method we use is evident; the PDR for the subscriber node with QoS 0 obtains an increase in PDR of between 64% (for 20 publisher nodes) and 23% (100 publisher nodes) as compared with the fixed RTO method used by MQTT-S. For the subscriber with QoS 1, this increase is between 76% (for 10 publisher nodes) and 21% (for 100 publisher nodes). As explained earlier, our method considers the SRTT as a network condition metric in order for it to react properly when a message loss occurs.

Although the MAC layer provides a reliability mechanism using MAC Acknowledgements, it is not sufficient to recover the message losses. As may be seen in Figure 6(b) below, although MAC Acknowledgements are used, the subscriber nodes with the RTO method used by MQTT-S obtain a lower PDR than in our method.

Therefore, with the use of our adaptive RTO method, the subscriber with QoS 0 achieves an increase in its PDR of between 38% (for 20 publisher nodes) and 12% (for 100 publisher nodes). For the subscriber node with QoS 1, the PDR increase is between 40% (for 20 publisher nodes) and 10% (for 100 publisher nodes).

**Figure 7.**
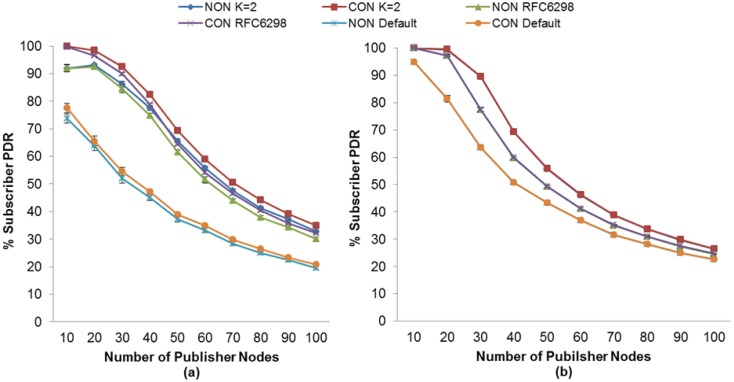
PDR comparisons between the RTO method of CoAP calculation and our proposal without (**a**) and with (**b**) MAC Acknowledgements.

The same situation occurs when we compare the PDR employing the RTO method used by CoAP with our approach, as may be seen in Figure 7. Although the initial RTO of CoAP is selected between 2 and 3 s and is doubled for consecutive retransmissions, unlike in our approach it is not sufficient to obtain a higher PDR.

In Figure 7(a), one may observe that without the use of MAC Acknowledgements the subscriber node with NON messages obtains an increase in its PDR of between 34% (for 30 publisher nodes) and 13% (for 100 publisher nodes) using our adaptive RTO method. In contrast, the increase in the PDR for a subscriber node with CON messages is between 38% (for 30 publisher nodes) and 14% (for 100 publisher nodes). Moreover, when using MAC Acknowledgements, the PDR of both subscriber nodes increases between 26% (for 30 publisher nodes) and 4% (for 100 publisher nodes), as shown in Figure 7(b).

Moreover, comparing our adaptive RTO method with CoAP using RFC6298 one may observe that, as showed in Figure 7(a) without the use of MAC Acknowledgment, both subscriber nodes get an increase in its PDR of between 5% (for 50 publisher nodes) and 3% (for 100 publisher nodes) using our adaptive RTO method. Moreover, when using MAC Acknowledgements, the PDR of both subscriber nodes increases between 13% (for 30 publisher nodes) and 1% (for 100 publisher nodes), as shown in Figure 7(b).

##### Comparison of RTT and RTO Measurements

**Figure 8.**
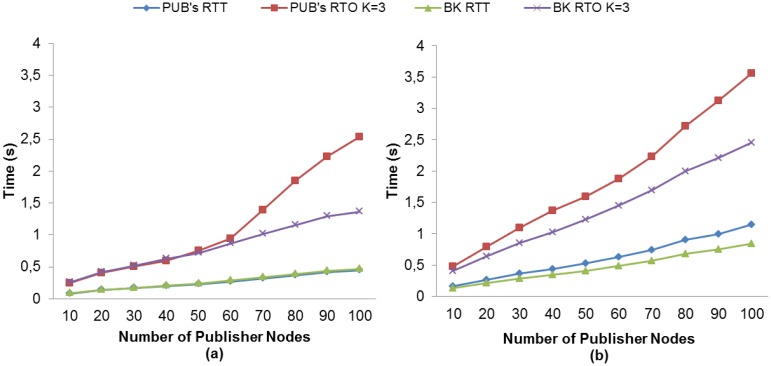
RTT and RTO comparisons for MQTT-S with our method without (**a**) and with (**b**) the use of MAC Acknowledgements.

We compare the measured RTT with the RTO calculated with our method to gain an insight into the behavior of the RTO as regards RTT. For MQTT-S, Figure 8(a) shows that without MAC Acknowledgements, the average RTT of publisher nodes is almost equal to that of the broker node, and in general the average RTO values are similar. The main difference occurs in the average RTO for publisher nodes, where this value is higher than for the broker node from 60 publisher nodes onwards. This is due to the fact that more publisher nodes are competing for access to the channel, and consequently the probability of packet collision increases. The RTO value therefore increases because the retransmissions from the application layer (MQTT-S) are activated. A similar situation is depicted in Figure 8(b). However, in this case a higher RTO value is obtained due to the use of MAC Acknowledgements.

**Figure 9.**
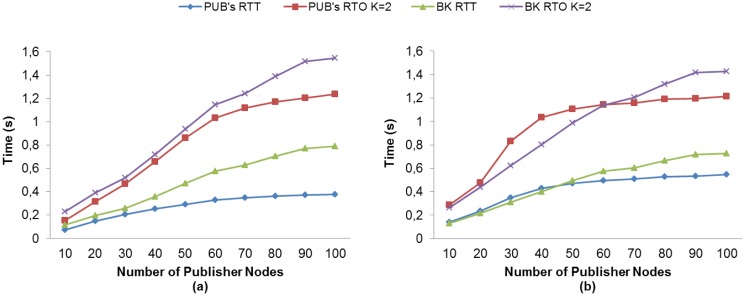
RTT and RTO comparisons for CoAP with our method without (**a**) and with (**b**) the use of MAC Acknowledgements.

On the other hand, Figure 9(a) shows the situation for CoAP without the use of MAC Acknowledgements. We can observe a change in the RTO behavior from 60 publisher nodes onward. The reason is the same as that previously explained for MQTT-S. However, the RTO value increases very slowly due to publication discipline of CoAP, which in turn results in the cancellation of retransmissions from the application layer (CoAP).

For the use of MAC Acknowledgements, the situation is the same, as illustrated in Figure 9(b), however, the change in the RTO behavior occurs from 40 publisher nodes onward. This difference is due to the fact that the use of MAC Acknowledgements causes the network to congest faster.

Comparison of the RTO for both protocols shows that, unlike MQTT-S, in CoAP the increase in the RTO value is very low from 60 publisher nodes onward. This is mainly due to the publication discipline of CoAP. In summary, the obtained RTO value with our adaptive RTO method adapts better than the fixed RTO method used by MQTT-S and CoAP.

#### 5.1.2. Discarded Publication Ratio (DPR)

**Figure 10.**
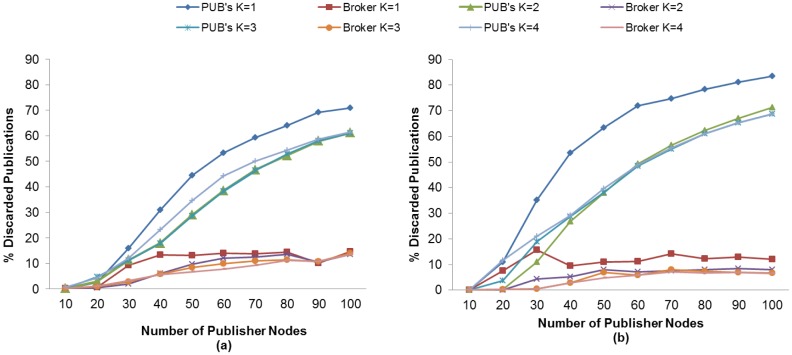
Effect of the number of Publisher nodes on the Discarded Publication Ratio from Broker and Publisher Nodes using different K values without (**a**) and with (**b**) MAC Acknowledgments.

**Figure 11.**
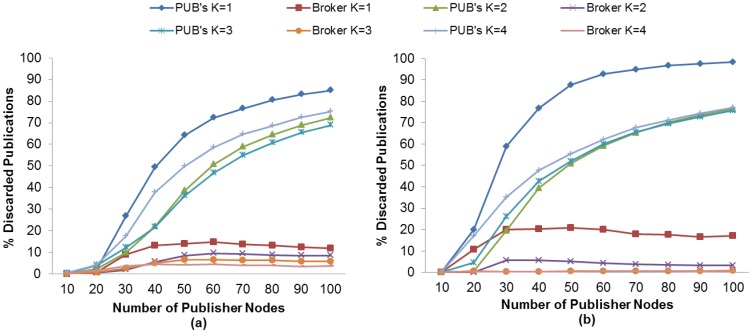
Effect of the number of Publisher nodes on the Dropped Publication Ratio from Broker and Publisher Nodes using different K values without (**a**) and with (**b**) MAC Acknowledgments.

The publication discipline of MQTT-S and CoAP has the effect of discarding publication messages, which in turn results in a reduction of the PDR. We evaluate the effect of the K value and the use of MAC Acknowledgements in the discarded publication ratio metric in order to find the setting with which we obtain the lowest DPR or match the highest PDR obtained in subscriber nodes. The results obtained show that for both protocols, MQTT-S and CoAP, publisher nodes have a higher discarded publication ratio than the broker node. This may be observed in Figures 10 and 11, and is mainly due to the fact that the publisher nodes do not receive the confirmation message from the broker node because of the loss of the publication message, the loss of the confirmation message, or because there is no channel access due to channel congestion. As a consequence, publisher nodes will carry out unnecessary retransmissions, thereby leading to an increase in the duplicated publications in the broker node. Moreover, MQTT-S will discard new publications, and CoAP will send a new publication and cancel possible retransmission of current one. The effect of retransmitted and duplicated messages is discussed in the following sections.

Regarding the effect of the K value, in both protocols we observe that DPR decreases in publisher nodes as the K value increases. However, our findings show that above a specific K value, the DPR increases. This specific K value depends on the protocol.

For MQTT-S in general, the DPR also decreases as the K value increases, except for K = 4. In this case, the retransmission of MQTT-S is activated too late to recover publication messages in the case of loss. This situation results in a higher DPR, since a new publication message could be generated while the RTO is activated, as can be seen in Figure 10. This situation occurs whether the MAC Acknowledgements is used or not.

In general, the lowest DPR for MQTT-S is obtained with K = 3 without the use of MAC Acknowledgements, as shown in Figure 10(a). However, Figure 10(b) shows that for a number of publisher nodes less than 40, we obtain the lowest DPR with K = 2 and using MAC Acknowledgements.

This is due to the increase in the number of publisher nodes, which in turn results in collision and loss of messages. For a number of publisher nodes less than 40, the value of K = 2 allows MAC Acknowledgements to recover most of the lost messages before MQTT-S retransmissions are activated from publisher nodes, thus the probability of generating a new publication message while the RTO is activated is reduced.

In contrast, a number of publisher nodes greater than 40 using MAC Acknowledgements gives rise to a higher probability of collision and loss of messages, and therefore an increase in delay. In this context, the value of K = 2 would lead to spurious retransmissions because the MQTT-S retransmissions would be activated before MAC Acknowledgements attempt to recover the lost messages. This situation would result in the discarding of publication messages due to a higher probability of generating a new publication message while RTO is activated.

Use of the value K = 3 enables most of the lost messages to be recovered before MQTT-S retransmissions are activated. Therefore, the probability of generating a new publication message while RTO is activated is reduced and so is the DPR. Finally, the setting using K = 3 matches the one that obtains the highest PDR for subscriber node with QoS 1, as seen previously in Figure 4(a).

A very similar situation occurs in the case of CoAP as that explained with MQTT-S. In general, the publisher nodes obtain a lower DPR as the K value increases, except for values of K above K = 2, as seen in Figure 11.

Regarding the use of MAC Acknowledgements, Figure 11(a) shows that the publisher nodes obtain a lower DPR without using MAC Acknowledgements than when using it; the absence of MAC Acknowledgements reduces the delay in receiving messages. As a consequence, the probability of generating a new publication is also reduced, and the RTO is activated. In fact, the setting for the lowest DPR is obtained without MAC Acknowledgements or K = 2, except for a number of publisher nodes less than 30, as shown in Figure 11(a). In this case, the lowest DPR is obtained with the use of MAC Acknowledgements, as can be seen in Figure 11(b). Moreover, this setting also matches the one that obtains the highest PDR for the subscriber node with CON messages, as seen previously in Figure 5(a).

In summary, the highest DPR occurs in publisher nodes for both protocols, as pointed out previously. Nevertheless, we have observed that the CoAP protocol obtains a higher DPR than MQTT-S protocol, based on the setting for the highest PDR of the subscriber node for each protocol. This situation is more evident for a number of publisher nodes greater than 30, as shown in Figures 10(a) and 11(a), and is due to the fact that an increase in the number of publisher nodes results in an increase in delay when sending the messages. Therefore, the probability of generating a new publication while the RTO is activated is greater, which results in the cancellation of the possible retransmission of the publication with a pending confirmation message and the transmission of the new one.

#### 5.1.3. Retransmitted Publication Ratio

The RTO value plays an important role in the number of retransmitted messages. As previously mentioned, an unsuitable RTO value would give rise to spurious retransmissions as well as the inability to react in time to recover message losses, which in turn results in a lower PDR. We therefore evaluate the K value effect and the use of MAC Acknowledgments in the retransmitted publication ratio in order to find the setting with which to match the highest PDR obtained in subscriber nodes discussed in an earlier section.

**Figure 12.**
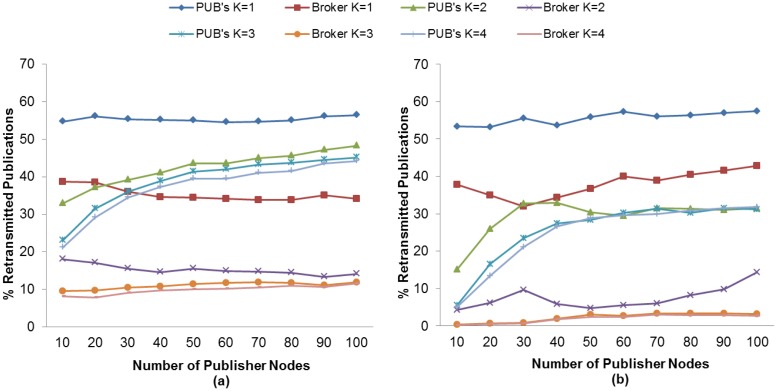
Effect of the number of Publisher nodes on publication messages retransmitted from Broker and Publisher Nodes using different K values without (**a**) and with (**b**) MAC Acknowledgments.

**Figure 13.**
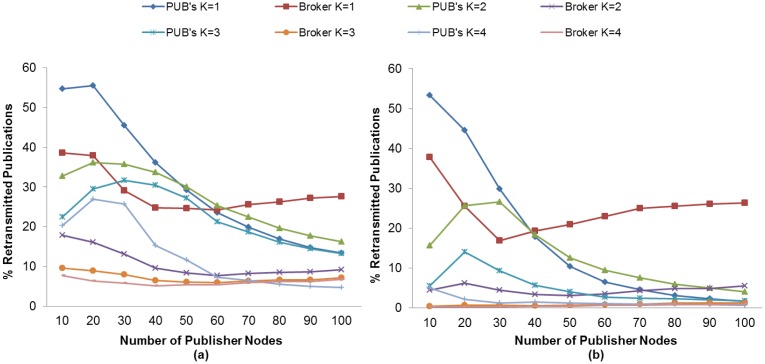
Effect of the number of Publisher nodes on publication messages retransmitted from Broker and Publisher Nodes using different K values without (**a**) and with (**b**) MAC Acknowledgments.

Figures 12 and 13 show the retransmitted messages ratio for MQTT-S and CoAP, respectively. It can be seen from these results that the lowest number of retransmitted messages is obtained with K = 4 with the use of MAC Acknowledgements. As expected, this is due to the decrease in the number of spurious retransmissions and the message recovery caused by the application layer retransmissions (MQTT-S and CoAP respectively) and the use of MAC Acknowledgements, respectively.

Nevertheless, neither MQTT-S nor CoAP obtain the highest PDR for subscriber nodes with this setting. As we have seen previously, for MQTT-S and CoAP with QoS 1, and the subscriber node with CON messages, we obtain the highest PDR without the use of MAC Acknowledgements with K = 3 and with K = 2, respectively. Comparison of both settings for MQTT-S in Figure 12(a) shows that although the number of retransmissions for K = 3 is higher than with K = 4, as illustrated in Figure 12(b), these number of retransmissions are necessary to recover the publication message in order to obtain the highest PDR.

A similar situation occurs with the CoAP protocol, as can be observed in Figure 13(a) for K = 2 and Figure 13(b) for K = 4, respectively. Although the setting with K = 4 enables the MAC layer to recover the publication message, this is insufficient.

For MQTT-S, the retransmissions are activated too late for packet recovery due to a larger RTO value. The DPR will therefore increase, because a higher probability of generating a new publication message exists, and the RTO is activated. Hence, the new publication message will be dropped and the PDR will decrease.

In the case of CoAP, the same situation occurs for K = 4. However, it should be pointed out that the decrease in PDR is due to the cancellation of possible retransmissions of publication messages. The publication messages with a confirmation pending will not be retransmitted in the case of loss, when a new publication is generated.

Finally, a comparison of MQTT-S with CoAP shows that the retransmitted message ratio for CoAP is lower than that for MQTT-S, as one may observe in Figures 12(a) and 13(a), respectively. This is because the publication discipline of CoAP always will cancel the potential retransmissions of a current publication message when a new publication is generated. This situation becomes more evident as the number of publisher nodes increases (starting from 30 publisher nodes), since a larger RTO value is generated due to the message delay caused by channel contention or message loss, as can be seen in Figure 13(a).

In summary, we have seen that publication retransmissions have a direct impact on the PDR, depending on the K value for the RTO calculation and the publication discipline.

#### 5.1.4. Duplicated Publications Ratio

Duplicated messages would reduce the PDR at the subscriber node because it receives useless data. In this context, we evaluate the effect of the K value and MAC Acknowledgment on the duplicated publication ratio to find the setting that matches the highest obtained PDR at subscriber nodes.

As expected, an inversely proportional relationship exists between the value of K and the number of received duplicated messages. As the value of K increases, the number of duplicated messages decreases, which in turn may cause a delayed reaction to packet recovery, especially when MAC Acknowledgements are not used. Otherwise, the number of duplicated messages increases due to spurious retransmissions. Both protocols show a similar behavior in terms of retransmitted messages.

**Figure 14.**
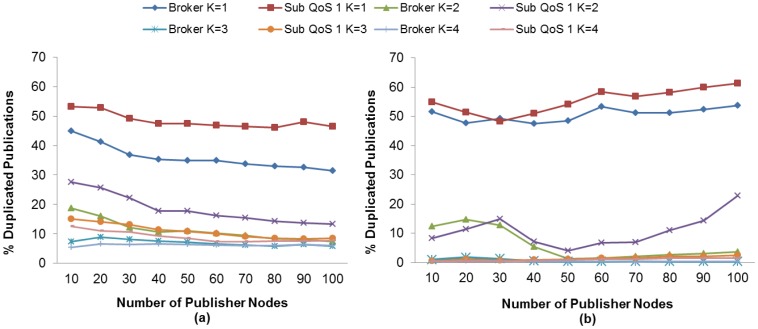
Effect of the number of Publisher nodes on the Duplicated Publications on Broker and Publisher Nodes depending on the K value of for RTO without (**a**) and with (**b**) MAC Acknowledgments.

**Figure 15.**
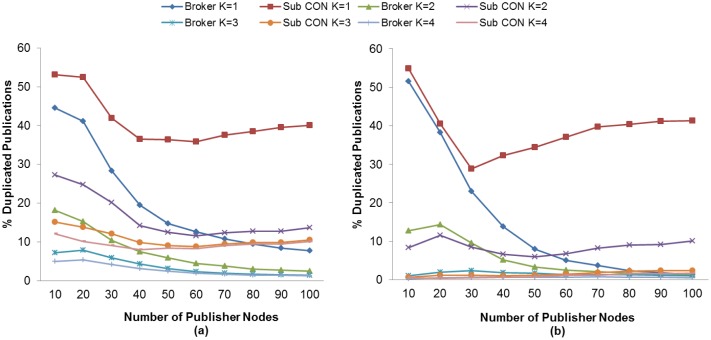
Effect of the number of Publisher nodes on the Duplicated Publications on Broker and Subscriber Nodes depending on the K value of for RTO without (**a**) and with (**b**) MAC Acknowledgments.

In this context, Figures 14 and 15 show that both the subscriber nodes and the broker node have the lowest ratio of duplicated messages with K = 4 and using MAC Acknowledgements.

However, we have already seen that the highest PDR is obtained without the use of MAC Acknowledgements and with K = 3 for MQTT-S and with K = 2 for CoAP, respectively. In this context, subscriber and broker nodes receive more duplicated publication messages, as shown in Figures 14(a) and 15(a). This is because MQTT-S and CoAP react faster in the case of loss of a publication message, and consequently spurious retransmissions may be produced.

In effect, the results show that for the MQTT-S protocol with K = 3 without MAC Acknowledgements, most of the duplicated messages received in the broker node are caused by spurious retransmissions from the publisher nodes, as shown in Figure 14(a). From 20 publisher nodes upwards, most retransmissions are necessary for recovery of publication messages. In fact, for MQTT-S, the duplicated message ratio decreases to approximately 6% from a number of publisher nodes greater than 20.

The situation is slightly different in the subscriber node with QoS 1. Figures 12(a) and 14(a) show that duplicated messages decrease to 8%, while retransmitted messages increase to 45%. One reason for this is that most of the duplicated messages are caused by spurious retransmissions from the broker node. The number of duplicated messages decreases proportionally as the number of publisher nodes increases in the network.

In the case of CoAP, most of the duplicated messages received in broker node are caused by spurious retransmission, as shown in Figure 15(a). Furthermore, the duplicated message ratio in broker node decreases as the retransmitted message ratio increases. This is more evident from 60 publisher nodes upwards. The reason for this is that an increase in the number of publisher nodes in the network leads to a higher probability of collisions and an increment in the contention for channel access, a situation that generates a greater delay. This means the messages may be lost or sent with a delay, in which case the RTO from publisher nodes is activated.

Due to the CoAP publication discipline, the number of retransmitted messages will decrease because a larger RTO value leads to higher probability of generating a new publication message when the RTO is active. A similar situation occurs with the subscriber with CON messages.

Finally, one may observe that with CoAP we receive fewer duplicated messages than with MQTT-S in the subscriber node with CON. The reason behind this situation is the difference in the publication discipline of both protocols that we discussed previously.

#### 5.1.5. Single-Hop Extended Scenario

**Figure 16.**
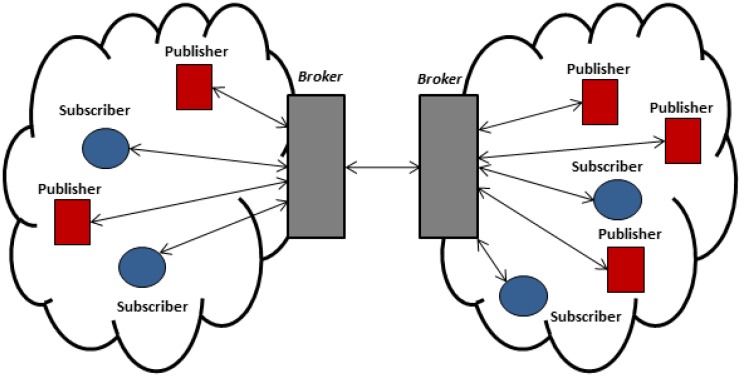
General View of Single-Hop Extended Scenario.

We extended the single-hop scenario shown in the previous section to build a distributed system based on two brokers as shown in Figure 16.

For this scenario, we observe that the behavior of K value is still the same as above scenario. That is, for MQTT-S the best value was K = 3 and for CoAP this value was K = 2 to obtain the highest PDR for subscriber nodes. Besides, as we expected, the obtained PDR was lower than the one in the single-hop scenario. For MQTT-S the PDR for subscriber node with QoS 0 was 2% (for 10 nodes) and 22% (40 nodes) lower than single-hop scenario. For the subscriber node with QoS 1 the PDR was between 2% (10 nodes) and 24% (40 nodes) lower. For CoAP the PDR for subscriber with NON messages was up to 2% (10 nodes) and 26% (40 nodes) lower than the single-hop scenario. Besides, the subscriber with CON messages gets a PDR 2% (10 nodes) and 24% (40 nodes) lower. The reason of that is because the addition of a second broker increases the network load which in turn results in packet losses. Besides, the messages between the broker nodes are sent in QoS 1. Therefore, for one of the broker nodes, the other broker node behaves as another subscriber with QoS 1. This situation results in more congestion, thus increasing of packet losses. Furthermore, the network load obtained with 70 publisher nodes is similar to the one obtained for 100 publisher nodes in single hop scenario. This is due to the increase network traffic caused by the second broker node. For this reason the result are showed up to 70 publisher nodes.

**Figure 17.**
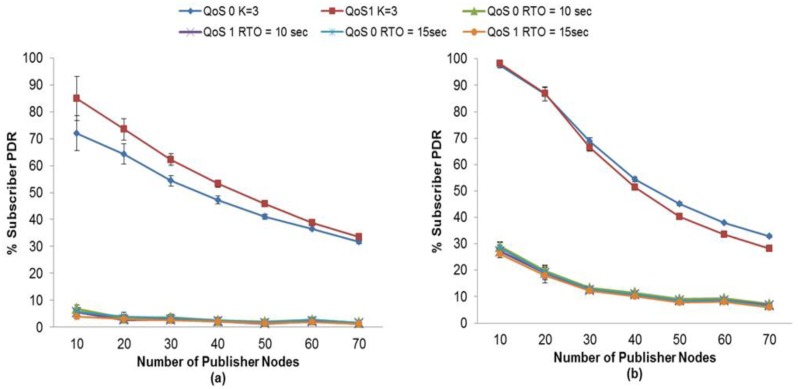
PDR comparisons between the RTO method of MQTT-S calculation and our proposal without (**a**) and with (**b**) MAC Acknowledgements.

However, regarding the use of MAC Acknowledgment, the results show different behavior depending on the protocol. In this context, for MQTT-S, the subscriber node with QoS 0 gets a higher PDR with the use of MAC Acknowledgment as shown in Figure 17(a). This is because the MAC Acknowledgments allow recovering the most of packet losses. In contrast, the subscriber node with QoS 1 gets the highest PDR without the use of MAC Acknowledgment. Nevertheless, Figure 17(b) shows that for a number of publisher nodes less than 40, the use of MAC Acknowledgment is required. The reason of this situation is because MAC Acknowledgment can recover the lost packets in situations of low traffic (up to 40 nodes). However, after this number of publisher nodes, the MAC Acknowledgments would congest the network faster resulting in an increase in packet delay and also in packet losses. This situation leads to an increase in probability to receive a new publication message from the application layer, while waiting for the ACK of an already sent one, which results in higher number of discarded publications. In this situation, the reliability mechanism of MQTT-S with K = 3 is the best among the ones evaluated to recover from packet losses without MAC Acknowledgment to get the highest PDR.

On the other hand, comparing the PDR obtained with our adaptive RTO method and the fixed RTO method we see that in MQTT-S, for the subscriber node with QoS 0, the PDR increase is between 69% (10 nodes) and 26% (70 nodes). In the case of subscriber node with QoS 1, the PDR increase is between 71% (10 nodes) and 27% (70 nodes). This demonstrates that the MQTT-S fixed RTO method is not suited to react to packet losses in this situation.

**Figure 18.**
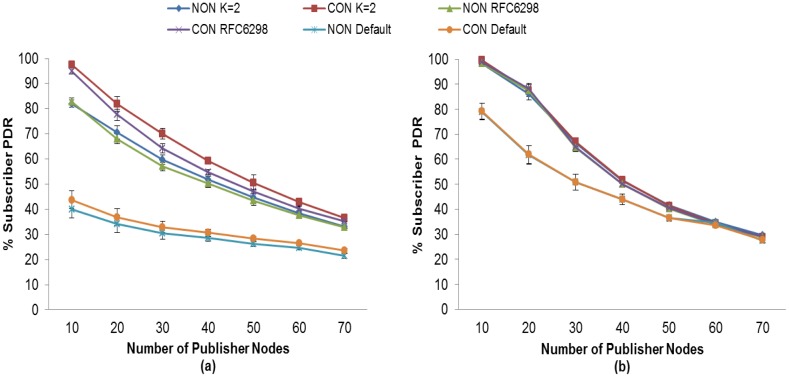
PDR comparisons between the RTO method of MQTT-S calculation and our proposal without (**a**) and with (**b**) MAC Acknowledgements.

For CoAP, we found that both subscriber nodes obtain the highest PDR without MAC Acknowledgements, as shown in Figure 18(a). Nevertheless, Figure 18(b) shows that for a number of publisher nodes less than 40, the use of MAC Acknowledgements is required for obtaining the highest PDR for a subscriber with NON messages. A similar situation applies for the subscriber with CON messages. In this case, the use of MAC Acknowledgements is required for a number of publisher nodes less than 30 in order to obtain the highest PDR. The reasons for this are the same as those previously explained for the subscriber node with QoS 1 using the MQTT-S protocol with our proposed RTO method.

On the other hand, the benefits for the subscriber node with NON messages using our RTO method is between 25% (20 nodes) and 4% (70 nodes) more than using the CoAP fixed RTO method as can be seen in Figure 18(a). In the case of subscriber node with CON messages this increase is between 25% (10 nodes) and 9% (70 nodes) shown in Figure 18(a). Besides, comparing CoAP using our RTO adaptive method and the one using RFC 6298, we observe that subscriber node with NON messages gets up to 2% more PDR using our adaptive RTO method. For the same comparison, the subscriber node with CON messages, the increase of PDR is around 5% as can be seen in Figure 18(b). The reason of this is that using RFC 6298 RTO method the publisher nodes discards between 2% to 5% more publication messages than our adaptive RTO method. Besides, the duplicated messages ratio increases around 3% to 10% more than our adaptive RTO method.

In the case of the retransmission publication ratio, this ratio decreases as the K value decreases. With K = 4 we get the lowest retransmission publication ratio and with use of MAC Acknowledgment. As expected, this is due to the application layer retransmissions (MQTT-S and CoAP respectively) and the use of MAC Acknowledgements decreases the number of spurious retransmissions and the message recovery. However, with this value the PDR for MQTT-S and CoAP decreases. This situation demonstrates that although the number of retransmissions for K = 3 for MQTT-S and K = 2 for CoAP is higher than with K = 4, these number of retransmissions are necessary to recover the publication message in order to obtain the highest PDR. The K value for the duplicated message ratio and discarded publication ratio (DPR) shows the same behavior than the retransmitted publication ratio we have explained.

Finally, we have studied the relation of the energy consumption of the nodes with the K value. As we expected, as the K value increases, the nodes energy consumption is decreased. For both protocols, the lowest energy consumption of the nodes is obtained with K = 4 and we also get the lowest retransmitted publication ratio. However, for this K value we obtain a lower PDR. That is, for MQTT-S the PDR decreases up to 5% for subscriber with QoS 0 and for subscriber with QoS 1 obtains up to 3% less PDR. In case of CoAP, the subscriber node with NON messages gets up to10% less PDR and for subscriber node with CON messages this decrease is up to 5%.

Moreover we have compared the energy consumption of our adaptive RTO method with the MQTT-S and CoAP. The results showed that with the use of the RTO methods of MQTT-S and CoAP the nodes consume up to 8% less energy compared with our RTO method, which creates a trade-off between energy consumption and the PDR.

Based on an overall comparison between MQTT-S and CoAP approaches, we see that the maximum achieved PDR by CoAP is better than that is achieved by MQTT-S. This is related to the publication discipline used. If the PDR of reliable node's delivery is the objective of an application our findings propose the use of CoAP.

### 5.2. Multi-Hop Scenario

**Figure 19.**
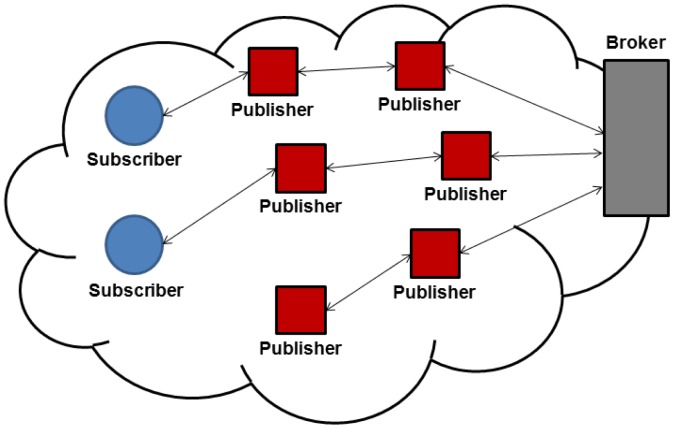
General View of Multi-Hop Extended Scenario.

This multi-hop scenario consists of nodes that are located up to 3 hops from the broker node as shown in Figure 19. The originating messages from a publisher node are routed to the broker node through multiple nodes. In a similar way, the received messages by the broker node are routed to the subscriber nodes.

For this scenario, the highest PDR for nodes was obtained using different K values compared with previous scenarios for both protocols. Besides, we can see that for both protocols the network load with 40 publisher nodes is very similar to the one with 100 publisher nodes in single-hop scenario. Therefore, we have obtained the results up to 40 publisher nodes.

**Figure 20.**
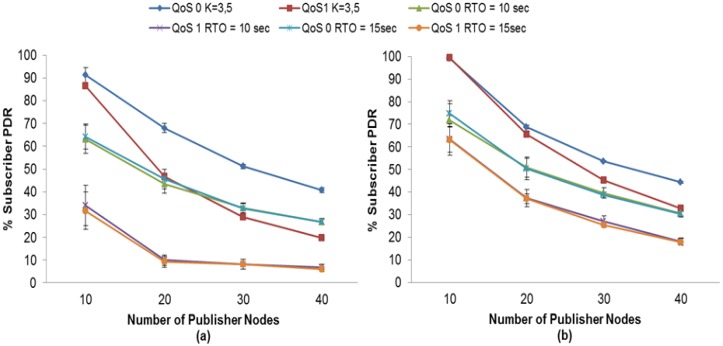
PDR comparisons between the RTO method of MQTT-S calculation and our proposal without (**a**) and with (**b**) MAC Acknowledgements.

For MQTT-S both subscriber nodes get the highest PDR with value of K = 3.5 and using MAC Acknowledgments as shown in Figure 20(b). The reason is the increase in RTT and the increase in packet losses caused by the different link conditions on each hop in the route to the destination. Therefore the use of MAC Acknowledgment is necessary to recover most of the packet losses on each hop. Moreover the value of K = 3.5 is proper to react to packet losses in situations where MAC Acknowledgments are not sufficient to recover from packet losses.

**Figure 21.**
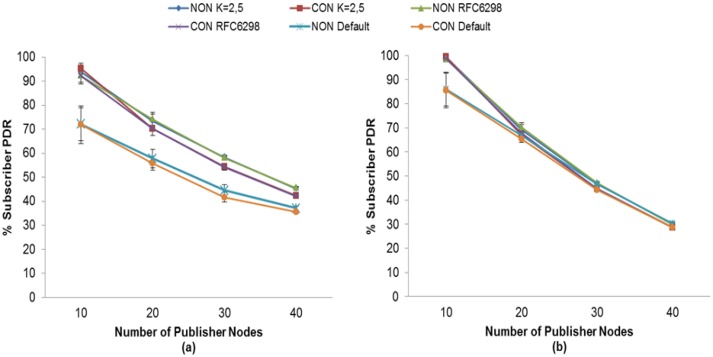
PDR comparisons between the RTO method of CoAP calculation and our proposal without (**a**) and with (**b**) MAC Acknowledgements.

In the case of CoAP, with K = 2.5 both subscriber nodes get the highest PDR without the use of MAC Acknowledgments as can be seen in Figure 21(a). The reason is that without the use of MAC Acknowledgments, the reliability mechanism of CoAP can react properly in case of packet losses for this K value. Otherwise, the use of MAC Acknowledgements leads to an increase of the message delay. In this situation, the value of K = 2.5 would result in spurious retransmissions because the CoAP retransmissions would be activated before MAC Acknowledgements attempt to recover the lost messages. This situation results in a low PDR for this subscriber node.

We also compared the PDR obtained with our adaptive RTO method and the fixed RTO method. The results show that for MQTT-S, the subscriber node with QoS 0, the PDR increase is between 27% (10 nodes) and 145 (40 nodes). For subscriber node with QoS 1, this increase is between 15% (10 nodes) and 36% (40 nodes). This demonstrates that the MQTT-S fixed RTO method using a value of with 10 and 15 s is not suited to react to packet losses in this scenario.

For CoAP, the benefits for the subscriber node with NON messages using our RTO method is between 7% (20 nodes) and 15% (40 nodes) increase in PDR compared to the fixed RTO method used by CoAP. The PDR for the subscriber node with CON messages obtains an increase of between 5% (20 nodes) and 14% (40 nodes) as compared with the fixed RTO method used by CoAP.

Besides, comparing CoAP using our RTO adaptive method and the one using RFC 6298, we observe that subscriber node with NON messages gets up to 2% more PDR using our adaptive RTO method. For the same comparison, the subscriber node with CON messages, the PDR increase is around 5%. The reason of this is that using RFC 6298 RTO method the publisher nodes discards between 2% to 5% more publication messages than our adaptive RTO method. Besides the duplicated messages ratio increases around 3% to 10% more than our adaptive RTO method.

For the other calculated metrics, we discuss the most important results to justify the reason because the subscriber nodes of both protocols get the highest PDR with K values, respectively. In the case of retransmitted publication ratio, the relation between the K value and the retransmitted publication ratio is the same as we have explained for previous scenarios. However, for MQTT-S, we get the lowest ratio with the use of MAC Acknowledgments, this is the reason because the subscriber nodes get the highest PDR with K = 3.5. In the case of CoAP, although the retransmitted publication ratio is lowest with the use of MAC Acknowledgments this is not very relevant to get the highest PDR. On the other hand, for discarded publication ratio (DPR) the results show that for CoAP, with K = 2.5 we get the lowest DPR on publisher nodes without the use of MAC Acknowledgments. This is because the subscriber nodes get the highest PDR without MAC Acknowledgments. In the case of MQTT-S, we get a lower DPR without MAC Acknowledgments, but this is not very significant to get the highest PDR.

Regarding the energy consumption, the results show the same behavior as in the previous scenario. That is, the energy consumption increases with the increase of retransmitted publication ratio. Regarding the energy consumption of our adaptive RTO method compared with the MQTT-S and CoAP, we find that the nodes consume in this scenario up to 6% less energy compared with our method, while losing from the PDR performance. It can be noted that our RTO method consumes only 1% more energy than the one on RFC 6298 used by CoAP.

Moreover, as we have showed for the studied scenarios, with all of these methods, the subscriber nodes achieve less PDR than our adaptive RTO method. This situation results in a trade-off between PDR and energy consumption. Therefore, we suggest that in applications in which PDR is not a critical requirement but the energy saving is very important, the choice of the RTO method should be considered.

Based on an overall comparison between MQTT-S and CoAP approaches, we observe that CoAP gets the maximum achieved PDR better than that is achieved by MQTT-S. This is related to the maximum achieved PDR by CoAP is better than that is achieved by MQTT-S. This is related to the publication discipline used. Our findings propose the use of CoAP in case of the PDR of reliable node's delivery is the objective of the application.

Finally taking into account the metrics we have discussed in this section we could design a mechanism to adapt the K value to the network conditions. The broker nodes could inform to publisher and subscriber nodes the K value to be used through piggybacked information in the confirmation or publication messages. The broker node begins with an initial K value, which can be adapted depending on duplicated publication ratio, DPR and retransmitted publication ratio we have discussed previously. Also the broker should consider the receiving message rate, the number of publisher nodes to calculate the network load and also the number of hops to the destination.

## 6. Conclusions

In this article, we describe and discuss the publish/subscribe interaction model of MQTT-S and CoAP protocols, which is the object of an increasing interest from the scientific community. We argue that these protocols use a reliability mechanism based on a fixed retransmission timer without considering any network metric. An adaptive RTO calculation method is more suitable in order to react properly to changing network conditions. We propose and evaluate an adaptive RTO method using the SRTT measurement and a K factor. We evaluate three different scenarios: single-hop, single-hop extended and multi-hop scenarios, for which we perform simulations for the RTO methods used by MQTT-S, CoAP and our proposal, along with the MAC Acknowledgment option for ensuring the one hop packet delivery. The results show that for the single hop scenario the adaptive RTO method we use provides an increase in PDR of between 64% (for 20 publisher nodes) and 23% (100 publisher nodes) for the subscriber node with QoS 0 as against the fixed RTO method used by MQTT-S. For the subscriber with QoS 1, this increase is between 76% (for 10 publisher nodes) and 21% (for 100 publisher nodes). These results are obtained without the use of MAC Acknowledgements in either method. Furthermore, the adaptive RTO method using MAC Acknowledgements also is higher than the fixed RTO method for both protocols. In this context, for MQTT-S, the subscriber with QoS 0 obtains an increase of between 38% (for 20 publisher nodes) and 12% (for 100 publisher nodes). For subscriber nodes with QoS 1, the PDR increase is between 40% (for 20 publisher nodes) and 10% (for 100 publisher nodes).

For CoAP, on the other hand, without the use of MAC Acknowledgements, the subscriber node with NON obtains an increase in its PDR of between 34% (for 30 publisher nodes) and 13% (for 100 publisher nodes). In contrast, the increase in the PDR for the subscriber node with CON is between 38% (for 30 publisher nodes) and 14% (for 100 publisher nodes). In addition, with the use of MAC Acknowledgements, the PDR of both subscriber nodes shows an increase of between 26% (for 30 publisher nodes) and 4% (for 100 publisher nodes).

For the multi-hop scenario the results show that our adaptive RTO method still provides a higher PDR for subscriber nodes compared to the other RTO methods. For MQTT-S, the increase of PDR is up to 36% for the subscriber node with reliability communication (QoS1 or CON message). Meanwhile, for CoAP this increase is up to 14% also for the subscriber node with reliability communication.

Moreover, the results show the effect of the chosen K value is mainly on the packet delivery ratio and the discarded publication ratio. We find the setting for obtaining the highest PDR for subscriber nodes, mainly for the node receiving publication messages in reliable mode (QoS 1 and CON). In general, an increase in the K value yields a higher PDR and a lower discarded publication ratio. In fact, the highest PDR using our adaptive RTO is obtained with K = 3 and 2 for MQTT-S and CoAP, respectively for single hop scenario. Meanwhile for multi-hop scenario the value found was K = 3.5 and 2.5 for MQTT-and CoAP, respectively. This behavior of K value demonstrates that we can obtain an optimized K value for each scenario to adapt to network conditions.

However, it is also necessary to take into account that this could lead to spurious retransmissions, and thus to duplicated messages. We also evaluate the effect of using MAC Acknowledgements. We identify the situations in which MAC Acknowledgements are useful for packet recovery, and consequently obtain a reduction in application layer retransmissions. It also transpires that, for the lowest evaluated K value, the use of MAC Acknowledgements is not recommended because it may give rise to high network congestion and consequently to a decrease in the PDR of the subscriber nodes.

Finally, regarding the publication discipline of each protocol, one may observe that the non-persistent mode used by CoAP leads to a higher PDR than that in the persistent mode used by MQTT-S. This is due to the fact that the CoAP publication discipline gives priority to sending the new publications while MQTT-S attempts to retransmit the old ones, a situation which increases PDR when the publication messages are received by the subscriber node, but the confirmation is lost. The choice of the publication discipline depends on the application area.

Evaluation of the results shows that the RTO calculation plays an important role in all the metrics evaluated. There is a trade-off between PDR and the retransmitted message ratio, depending on the K value. Therefore, the paper demonstrates that we can achieve better performance by using the optimized K value. Based on the insight gained, for a future work, we propose the design of an algorithm to adapt the K value dynamically to the network conditions. It could be managed from the broker node where this node begins setting an initial K value and then this value would be adapted depending on duplicated publication ratio, DPR and retransmitted publication ratio metrics we have discussed previously. Also the broker node should consider the receiving message rate, the number of publisher nodes to calculate the network load and also the number of hops to the destination. We thereby expect to gain control of duplicated messages that lead mainly to energy waste, thus increasing the PDR of subscriber nodes. This is the reason of the trade-off observed in the results of this study between PDR and energy consumption.

## Figures and Tables

**Table 1.** CoAP protocol constants for message transmission.

**Protocol Constants**	**Value**
ACK_TIMEOUT	2 s
MAX_RETRANSMIT	4
ACK_RANDOM_FACTOR	1.5

**Table 2.** Parameter setting for simulation.

**Parameter**	**Value**
Carrier Frequency	2.4 GHz
Bit rate	250 kbps
Max. number of retransmissions in App. layer	4
Max. number of retransmissions in MAC layer	3 (default for IEEE 802.15.4)
Publication generation interval	1 second
Publication message size	74 bytes
Traffic type	Periodic
